# Association between Types of Screen Time and Weight Status during the COVID-19 Pandemic: A Longitudinal Study in Children and Adolescents

**DOI:** 10.3390/nu15092055

**Published:** 2023-04-24

**Authors:** Yujie Liu, Xiaomin Sun, Erliang Zhang, Huilun Li, Xin Ge, Fan Hu, Yong Cai, Mi Xiang

**Affiliations:** 1 International Peace Maternity and Child Health Hospital, School of Medicine, Shanghai Jiao Tong University, South Chongqing Road No. 227, Shanghai 200025, China; 2 School of Public Health, Shanghai Jiao Tong University, Shanghai 200025, China; 3 Global Health Institute, School of Public Health, Xi’an Jiaotong University Health Science Center, Xi’an 710061, China

**Keywords:** screen time, obesity, children and adolescents

## Abstract

Background: This study aimed to investigate the changes in distinct types of screen time and explore their longitudinal association with children and adolescents’ weight status. Methods: A two-wave longitudinal study was conducted among 2228 children and adolescents (6–19 years) in Shanghai, China, before and during the pandemic. Recreational screen time (watching TV/videos, online gaming, using social media, and browsing webpages), educational screen time (online homework and online class), and BMI were measured using a self-reported questionnaire. Mixed-effects models were constructed to assess the associations between screen time and weight status. Results: The prevalence of overweight and obesity was 20.5% and 10.2% at baseline, respectively. Both recreational and educational screen time increased significantly over two months. While recreational screen time was found to be a risk factor for obesity, it was not the case for educational screen use. Specifically, adolescents who spent more time watching TV/videos had a higher obesity risk (OR = 1.576). No significant associations were found in children. Conclusions: Overweight and obesity were prevalent among children and adolescents in China. Reducing screen-based activities is a promising strategy to prevent unhealthy weight gain in Chinese children and adolescents, while it is necessary to consider the content and distinguish between educational and recreational screen use.

## 1. Introduction

Overweight and obesity (ow/ob) among children and adolescents has emerged as one serious public health problem worldwide, with the global prevalence of pediatric obesity increasing significantly from 0.7% to 5.6% for girls and from 0.9% to 7.8% for boys over the past few decades [1]. Increased obesity rates have also been reported among children and adolescents in China [2]. The latest national data from 2015 to 2019 show that 7.9% of children and adolescents aged 6–17 years in China are living with obesity, and another 11.1% are classified as overweight [3]. Ow/ob in children and adolescents can result in an abundance of adverse health problems, including metabolic disorders, cardiovascular abnormalities, inflammation, and psychological disorders. The onset of those disorders may begin in childhood and adolescence and track into adulthood [4]. In addition, in the face of the COVID-19 pandemic, governmental measures such as home confinement and school closure have significantly increased risks of ow/ob by limiting their participation in regular individual or group physical activities outside [5,6]. Reports from China documented a significant increase in weight gain among youth compared with the pre-pandemic rate, with the prevalence of obesity increasing from 10.5% to 12.6% [7].

Lifestyle behaviors are key risk factors for children and adolescents ow/ob [8,9]. Previous studies have suggested that excessive screen time increases the risk of ow/ob among children and adolescents, with more research focused on the impact of recreational screen time [10,11]. However, in addition to the well-studied recreational screen use, a significant portion of screen time is dedicated to learning-related activities (e.g., online courses and homework), especially for students in Asian countries like China who face heavy academic pressure [12,13]. There is growing evidence that the health effects of screen use are contingent upon the type of screen activity [14,15,16]. For example, Sanders found that educational screen use has no negative associations with physical health outcomes, while recreational screen use is linked to adverse outcomes among children [14]. Therefore, it is plausible to suggest that the associations between screen use and child ow/ob may also vary for the specific type of screen use behavior. Furthermore, most of the previous studies on screen time were based on cross-sectional design [17,18,19]. Given the significant impact of the COVID-19 pandemic on daily routines when children and adolescents are confined to their homes [5,6], the negative health effects of excessive screen use are likely to be even more severe. To fill the gap in current research, it is crucial to explore the longitudinal associations between specific screen use behaviors and weight status among children and adolescents during this unique period of dramatic environmental changes.

Using longitudinal data collected from students and their parents in Shanghai, China, before and during the COVID-19 outbreak, the present study aimed to assess: (1) changes in the different screen use behaviors of children and adolescents before and during the pandemic; and (2) longitudinal associations between specific types of screen time with children and adolescents’ weight status.

## 2. Materials and Methods

### 2.1. Study Design and Study Sample

An online longitudinal study among children and adolescents aged 6–19 years in Shanghai, China, was conducted. Seven districts in Shanghai were first designated (14 districts in total), then 1–2 schools from each district were selected via cluster sampling. Participants from ten primary or junior high schools were recruited in the first survey, and five of them completed the second survey. All children and adolescents and their parents in the five selected schools were invited to participate. Written informed consent was obtained from the parents before the survey.

**Figure 1:**
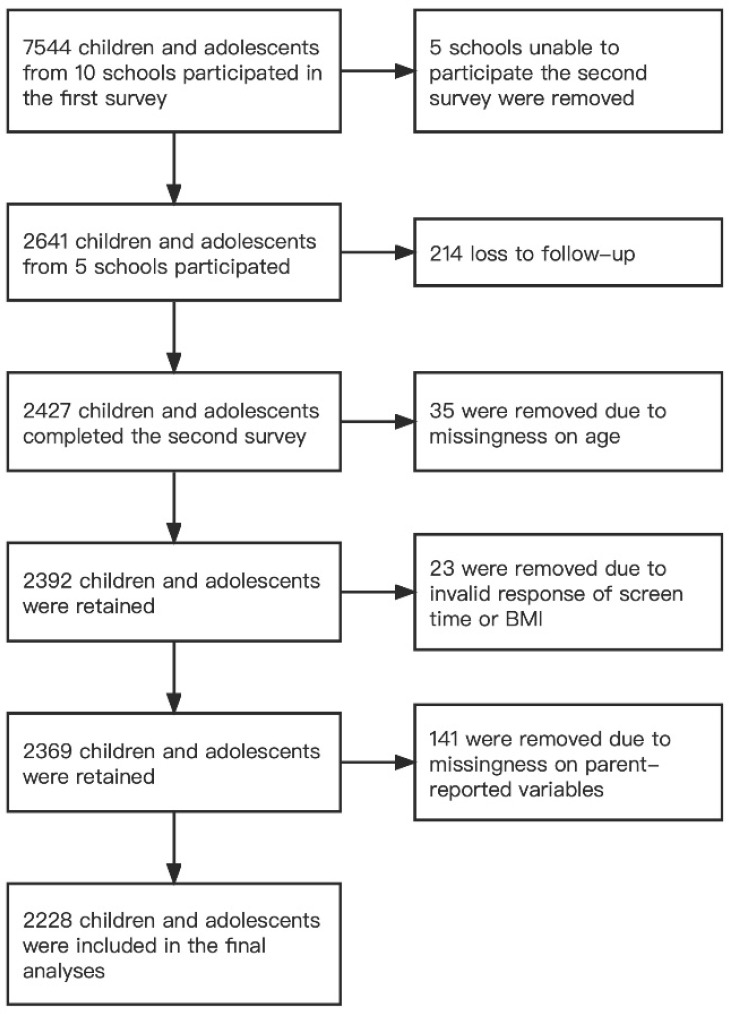
Flow of participants.

The first survey recruited 2641 children and adolescents in the five schools between 3 and 21 January 2020 (before the COVID-19 outbreak). A Level 1 public health emergency was subsequently declared in Shanghai on 24 January 2020 [20]. The government ordered citizens to stay at home and implemented school closures to prevent the further spread of the infection. Children and adolescents were confined at home and took online classes during this period. Approximately two months after the COVID-19 outbreak, the second survey was conducted between 13 and 23 March 2020 to 2427 children and adolescents who completed the first survey. Shortly after, the public health emergency response was downgraded from level 1 to level 2 in Shanghai on 24 March 2020 [21]. The strict lockdown was ended, and schools reopened subsequently. Details are provided in Table S1. Participants with missing or invalid data on age, weight, height, screen time, or parent-reported variables were excluded, and a total of 2228 children and adolescents were included in the final analyses. A flow chart of participant exclusion is shown in Figure 1.

Parents were informed about the study goal, procedure, and rules. Their permission was obtained prior to the survey. The online questionnaire was distributed to parents and then to their children. The research members were asked to answer questions from students and parents concerning the surveys. The questionnaire consists of a section for students and another section for their parents, completed by students (children and adolescents) and parents separately. To address the possible recalling bias and comprehension difficulties in young children, primary school students completed the questionnaire under the instruction of their parents.

### 2.2. Key Study Variables and Measurements

#### 2.2.1. Outcome Variables

Body weight and height were reported by the children and adolescents in the two surveys. For primary school students, their parents were asked to assist in providing the height and weight data for accuracy. Then BMI was calculated by dividing self-reported weight by self-reported height squared (kg/m^2^). Ow/ob was defined based on the age- and sex-specific BMI standard developed by the World Health Organization (WHO) using the preferred standard deviation (SD) or z-score system: >1 (SD) represents overweight, and >2 SD represents obesity [22,23].

We compared the agreement of the self-reports with body height and weight measured by physical examination among 1107 children and adolescents in two schools in a pilot study. Intraclass correlation coefficients (ICC) were applied and showed moderate-to-excellent absolute agreement according to Koo & Li (r = 0.972 for height; r = 0.934 for weight; r = 0.717 for BMI) [24].

#### 2.2.2. Exposure Variables

Screen time was reported by the children and adolescents in the two surveys. Parents were instructed to assist primary school students in answering the questions. Screen use items were built on the different categories of screen use proposed by Sanders [14], as well as results derived from our pilot study about screen use, including recreational and educational items. Recreational screen use was further divided into (1) watching TV/videos, VCDs, and DVDs; (2) computer/smartphone gaming; (3) social media use (QQ, WeChat, etc.); and (4) browsing webpages (news, douban, etc.). Educational screen use included online homework and online class. Participants were first required to answer whether they were engaged in each screen behavior (“yes” or “no”). If “yes” was selected, then the number of days per week (0–5 for weekdays and 0–2 for weekends) and the average time per day of each screen behavior were reported on weekdays and weekends, respectively. The average daily screen time was calculated by averaging the time spent on weekdays (5 days) and weekends (2 days) screen time.

#### 2.2.3. Covariates

Household annual income (<200,000 yuan, 200,000–500,000 yuan, and >500,000 yuan), parents’ highest education level (middle school or below, high or vocational school, and college or above), and parents’ body weight and height were reported by the parents. Parents’ BMI was calculated using body weight divided by height squared (kg/m^2^) and included as confounders to adjust for genetic and family environmental effects. According to WHO, overweight and obesity in the adult population are defined as BMI ≥ 25 and BMI ≥ 30, respectively [25].

Physical activity levels and dietary patterns were reported by the children and adolescents. Parents were instructed to assist primary school students in answering the questions. Children’s moderate and vigorous-intensity physical activity (MVPA) participation was measured using the Global Physical Activity Questionnaire (GPAQ) developed by the WHO. The Chinese version of the GPAQ was employed, which has demonstrated acceptable reliability and validity in measuring MVPA [26]. Physical activity level was defined as inactive (<30 min/day), insufficiently active (30–60 min/day), and sufficiently active (≥60 min/day) according to the guidelines for children [27]. Dietary pattern was assessed by the Chinese version of a quantitative Food Frequency Questionnaire (FFQ) [28]. Total energy intakes were calculated using the FFQ based on the Chinese Food Composition Tables [29]. Physical activity level and total energy intake were also included as behavioral confounders.

### 2.3. Statistical Analysis

First, we described children’s characteristics, including sociodemographic variables, weight status, physical activity level, total energy intake, parents’ BMIs, and screen use behaviors using data collected at baseline. Chi-square tests were used to compare the differences between the children classified as having normal weight, overweight, and obesity for categorical variables and analysis of variance (ANOVA) for continuous variables. Multinomial logistic regressions were conducted to investigate the relationship between different types of screen use behaviors and child ow/ob (normal weight group served as the reference group).

The average daily time spent on overall and specific types of screen use was described in the 2 surveys, reported in minutes. T-tests were conducted to compare the differences in screen time from before to during the pandemic. Then mixed-effects models were fitted to assess the longitudinal associations between standardized screen use time (screen time transformed by converting the raw data to a standard format with mean 0 and standard deviation 1) and weight status. A series of models were constructed using obesity and BMI as dependent variables, respectively. In Model 1, children’s age and gender were adjusted. In Model 2, family covariates, including parents’ highest education levels and parents’ BMIs, were further adjusted. Other behavioral covariates, including physical activity level and total energy intake, were added in the final Model 3.

All statistical analyses were performed using R 3.6.1. A *p*-value less than 0.05 was considered significant.

### 2.4. Ethics

All procedures performed in studies involving human participants were in accordance with the 1964 Helsinki Declaration. The study protocol was approved by the Ethics Committee of Shanghai Jiaotong University School of Medicine (SJUPN-201813) on 8 March 2019.

## 3. Results

### 3.1. Sample Characteristics

Table 1 shows the characteristics of children and adolescents at baseline. Children and adolescents’ average BMIs were 19.0 ± 4.21 kg/m^2^. The prevalence rates of overweight and obesity were 20.5% and 10.2%, respectively. Boys had a significantly higher rate of ow/ob than girls (*p* < 0.001). Younger participants were more likely to live with obesity compared to their counterparts (*p* < 0.001). Children and adolescents with higher parents’ BMIs (*p* < 0.001) and lower physical activity levels (*p* = 0.003) had a higher risk of developing ow/ob.

Table 2 shows the descriptive statistics of different types of screen use at baseline and their associations with ow/ob. On average, participants spent 41.3 ± 58.3 and 82.7 ± 100.6 min per day on recreational and educational screen use. Results from the multinomial regression models showed that recreational screen time was significantly associated with weight status (*p* = 0.040). When screen use behaviors were further divided, children and adolescents who spent more time watching TV/videos (*p* = 0.038) and computer/smartphone gaming (*p* = 0.007) had a higher obesity risk.

### 3.2. The Dynamic Changes in Screen Use Behavior

**Figure 2:**
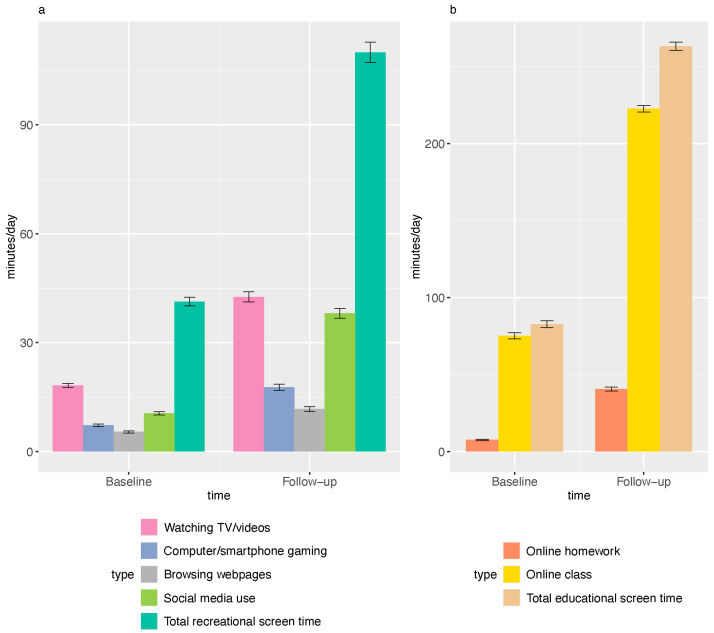
Changes in different types of recreational screen time (**a**) and educational screen time (**b**) among children and adolescents. Error bars indicate standard errors.

The time spent on recreational (mean ± SD, 41.3 ± 58.3 vs. 110.0 ± 132.3 min/day, *p* < 0.001) and educational screen use (mean ± SD, 82.7 ± 100.6 vs. 263.3 ± 127.6 min/day, *p* < 0.001) increased significantly during the COVID-19 pandemic. More specifically, the mean time in watching TV/videos (mean ± SD, 18.1 ± 25.7 vs. 42.6 ± 64.0 min/week, *p* < 0.001), computer/smartphone gaming (mean ± SD, 7.2 ± 17.3 vs. 17.7 ± 38.4 min/week, *p* < 0.001), social media use (mean ± SD, 10.5 ± 22.7 vs. 38.0 ± 63.7 min/week, *p* < 0.001), browsing webpages (mean ± SD, 5.4 ± 13.8 vs. 11.7 ± 29.7 min/week, *p* < 0.001), online homework (mean ± SD, 7.5 ± 22.3 vs. 40.6 ± 61.9 min/week, *p* < 0.001), and online classes (mean ± SD, 75.1 ± 96.4 vs. 222.7 ± 101.6 min/week, *p* < 0.001) all increased significantly (Figure 2).

### 3.3. Associations between Screen Time and Weight Status

Table 3 displays longitudinal associations between standardized screen time and obesity. When age, sex, and other family and behavioral factors were adjusted, recreational screen time was positively associated with obesity risk (OR = 1.518, 95% CI: 1.159–1.988). This result indicates that for each 1-SD increase in recreational screen time (107.7 min of unadjusted recreational screen time per day), the odds of developing obesity increase by approximately 52%. Among different types of recreational screen use, TV/video viewing was the only significant risk factor for obesity (OR = 1.576, 95% CI: 1.230–2.020). No significant associations were found between educational screen time and obesity risk. When stratified by age (child <10 years old and adolescent ≥10 years old), the associations of screen time with obesity were only observed in the adolescent group (OR = 1.470, 95% CI: 1.040–2.078 for recreational screen time; OR = 1.485, 95% CI: 1.080–2.042 for TV/video viewing) (Table 4).

Longitudinal associations between standardized screen use time and BMI are displayed in Table S2. In the final model adjusted for all confounders, a positive association was found for recreational screen time—the BMI increased by 0.071 for each 1-SD increase in recreational screen time. Specifically, participants who spent more time watching TV/video (β = 0.091, 95% CI = 0.033–0.148) showed higher BMI. No significant associations were found between educational screen time and BMI. When stratified by age, the associations of screen time with BMI were only observed in the adolescent group (β = 0.077, 95% CI: 0.009–0.145 for recreational screen time; β = 0.066, 95% CI: 0.003–0.130 for TV/video viewing) (Table S3).

## 4. Discussion

Using longitudinal data collected before and during the COVID-19 outbreak in Shanghai, China, the present study first explored the impacts of changes in different types of screen time on children and adolescents’ weight status in the context of dramatic social and environmental change. Lifestyles changed considerably as a result of the pandemic, increasing both the amount of time spent on educational and recreational screen use. Recreational screen time was longitudinally associated with weight status even after controlling for dietary intake, while this is not the case for educational screen time. Given that approximately 1/3 of the children and adolescents had ow/ob, reducing recreational screen time could be a promising strategy to prevent unhealthy weight status, which has significant implications for promoting child and adolescent health.

In the present study, screen time, including both recreational screen time (watching TV/videos, computer/smartphone gaming, social media use) and educational screen time (online homework and online courses), increased significantly during the COVID-19 pandemic. This is consistent with previous findings that when children are out of school, they tend to adopt unhealthy lifestyles with less physical activity and increased screen time [30]. The increase in educational screen time in this study was the result of the changes in learning patterns from offline to online courses, which significantly increased the reliance on electronic screens. In contrast to the necessity of completing learning tasks, the increase in recreational screen time is more alarming. During home confinement, children and adolescents spontaneously replaced outdoor activities with screen use as a primary form of entertainment, resulting in an increased risk of problematic internet use [31,32]. In addition, excessive recreational screen use can be habit-forming and continue to impose long-lasting detrimental effects. The further exacerbation of ow/ob problems in children and adolescents is one of the potential negative consequences of increased recreational screen time.

To date, accumulating cross-sectional studies have concluded that more screen time is associated with higher levels of obesity risk in children and adolescents. However, there is little longitudinal evidence for this association [33,34,35]. For example, in the National Institute of Child Health and Human Development Study of Early Child Care and Youth Development, change in BMI was positively associated with screen time at the upper tail of BMI distribution [36]. Another cohort study conducted in five major cities across China found that school children who spent more time on screen use had a higher risk of developing obesity [37]. One major limitation is that these longitudinal studies only investigated overall screen time while not distinguishing the sub-types. Therefore, it remains unclear whether different types of screen use could have the same health effects. Our study filled this gap by exploring the distinct associations of educational and recreational screen time with children and adolescents’ weight status. In the present study, weight status was associated with recreational screen time, while no significant association was observed for educational screen time. These results indicate that more attention should be paid to the portion of the total screen time children and adolescents spend on entertainment.

Minimizing screen use is an essential strategy for preventing and controlling childhood obesity [38]. However, duration is not the only dimension of screen use to be considered. The types may also lead to different health consequences. The present study further explored the various obesity risks associated with specific types of recreational screen use, revealing that prolonged TV/video viewing could significantly increase obesity risk. This is consistent with the previous findings that passive screen use, separate from more socially interactive forms (e.g., computer gaming), significantly affects childhood obesity [39,40]. One possible reason is that TV/video viewing facilitates access to commercial unhealthy food ads as well as possible over-eating [41]. However, the associations remained significant in our study even after controlling for total energy intake, indicating that dietary factors are not the sole explanation. Since children tend to be less focused during passive screen use, the negative impact on weight status may also arise from a lack of mental activity compared to active use. Similarly, no significant association was found in this study between weight status and educational screen time, which involves challenging mental tasks. This result is consistent with a previous finding that educational screen time is associated with positive educational outcomes and has no negative effect on weight-related physical health [14]. However, considering the other health problems (e.g., poor sleep, impaired vision, and psychological distress) associated with excessive educational screen use [42], it is still necessary for schools and parents to balance the potential health risks and educational benefits.

Another finding of our study was that the effects of screen time varied by age. This aligns with previous research that has indicated a significant association between recreational screen time, particularly TV viewing, and weight status among adolescents but not children [43,44]. Jakubec et al. suggest that this variation could be explained by a decline in family influence on health behaviors during the developmental shift from childhood to adolescence, whereby adolescents are less restricted by parents and more prone to unhealthy weight gain due to excessive screen use [45]. Despite the lack of a significant correlation between screen time and children’s weight status in this study, the potential effects of screen time on children cannot be disregarded entirely. Considering the high prevalence of ow/ob among children (18.6% for overweight and 12.6% for obesity), it is essential to exercise caution regarding the health risk associated with excessive screen use in this population.

In the present study, approximately 1/3 of the children and adolescents lived with ow/ob, which is significantly higher than the estimated prevalence of ow/ob in Chinese children and adolescents from 2015 to 2019 [3]. This result is in accordance with the growing rates of ow/ob among children and adolescents in recent years, which is recognized as a major public health problem. The widespread use of electronic devices in recent years is one of the main causes of the increase in ow/ob prevalence among children and adolescents. Within the long-term increasing trend, children and adolescents may experience additional increases in screen time as a result of life events like COVID-19. For example, short-term fluctuation in screen time may occur when children and adolescents are on holiday breaks from school, potentially increasing the risk of unhealthy weight gain [30]. Proactive preparations can be made to mitigate the impact of a similar situation in the future.

Our study has important practical implications in the face of the public health challenge of childhood obesity. First of all, limiting recreational screen use, especially TV or video viewing, can be a crucial point of early prevention for unhealthy weight status. Since school children tend to spend most of their leisure screen time out of school, families play an integral role in the efforts to reduce screen time. Evidence from previous studies has demonstrated the effectiveness of screen time intervention strategies which incorporated family involvement, parental support, and role modeling [46]. To reduce their children’s screen use, parents are encouraged to be good role models for healthy screen use. Other effective measures include setting clear rules on screen time, encouraging other non-screen-based activities, and having open conversations with their children about the negative effects of excessive screen use. In contrast, schools have a greater impact on educational screen use. Although no significant effect was found for educational screen use in this study, the potential health risk is still a matter of concern given its large proportion of the total screen time. Now that the pandemic has subsided, schools can prioritize offline classes and use online educational apps selectively. Since screen-based learning typically requires uninterrupted sedentary behaviors, it would be better to incorporate possible movement and break up the prolonged period of sedentary study [38].

The main strength of our study is the longitudinal investigation of changes in distinct types of screen time under a unique circumstance with dramatic lifestyle changes, providing natural experimental evidence on the associations between different screen use and children and adolescents’ weight status. Another strength is that multiple covariates, including parents’ BMI, physical activity level, and dietary intake, were included to control for confounding effects of genetic, family environmental, and behavioral.

Despite these strengths, the present study also has some limitations. First, data for this study was collected using self-reported questionnaires. Self-reported anthropometric data narrowed the possibility of generalizing the results due to recalling bias. In order to improve the accuracy and reliability of our results, we compared the self-reports with the body height and weight measured by a physical examination in a prior pilot study and found good agreement. Furthermore, the self-reported measures of screen time may result in social desirability bias and underestimate the actual data. Although any possible types of screen use behaviors were included in the questionnaire, the actual effects of screen time on weight status may be compromised due to the inherent bias. Therefore, objective methods to investigate screen time (e.g., accelerometer) and weight status (e.g., portable stadiometer and electronic weighing scales) among children and adolescents are warranted in future studies. Second, despite the large sample size, our study only investigated the prevalence of ow/ob in Shanghai, China. We should be cautious in generalizing the present findings to children and adolescents nationwide. Finally, the two-month follow-up period in this study was relatively short. However, significant changes in screen time were observed in this special study period.

## 5. Conclusions

This longitudinal study established the association between changes in different types of screen time and the weight status of children and adolescents during the COVID-19 pandemic. Both educational and recreational screen time increased dramatically in this two-month short period, which may lead to potential physical health impacts in the long term. In addition, adolescents who spent more time in recreational screen use had a higher obesity risk, while educational screen time was not a significant risk factor in this study. No significant associations were found in children. Our findings suggest that families and schools should focus on limiting screen-based activities, especially recreational activities, to prevent and control unhealthy weight status.

## Figures and Tables

**Table 1:** Sample characteristics at baseline.

Characteristics	Total (*n* = 2228)	Overweight/Obesity		
		Overweight (*n* = 468)	Obesity (*n* = 232)	*p*-Value *
BMI, mean (SD)	19.0 (4.21)	21.6 (2.31)	26.7 (5.03)	<0.001
Child sex, *n* (%)				
Male	1127 (50.6%)	303 (64.7%)	164 (70.7%)	<0.001
Female	1101 (49.4%)	165 (35.3%)	68 (29.3%)	
Child Age (in years), mean (SD)	10.9 (2.49)	11.2 (2.40)	10.5 (2.33)	<0.001
Child grade, *n* (%)				
1–3	535 (24.0%)	88 (18.8%)	66 (28.4%)	0.004
4–6	777 (34.9%)	182 (38.9%)	88 (37.9%)	
7–9	916 (41.1%)	198 (42.3%)	78 (33.6%)	
Father Obesity related variables, *n* (%)				
BMI (in kg m^−2^), mean (SD)	26.1(6.96)	26.3 (6.34)	28.9 (8.80)	<0.001
Normal	1255 (56.3%)	234 (50.0%)	86 (37.1%)	<0.001
Overweight	732 (32.9%)	184 (39.3%)	89 (38.4%)	
Obesity	241 (10.8%)	50 (10.7%)	57 (24.6%)	
Mother Obesity related variables, *n* (%)				
BMI (in kg m^−2^), mean (SD)	23.3 (6.20)	23.6 (5.93)	25.4 (7.24)	<0.001
Normal	1837 (82.5%)	376 (80.3%)	161 (69.4%)	<0.001
Overweight	230 (10.3%)	61 (13.0%)	46 (19.8%)	
Obesity	161 (7.2%)	31 (6.6%)	25 (10.8%)	
Household income, *n* (%)				
<100,000 yuan	262 (11.8%)	46 (9.8%)	39 (16.8%)	0.204
100,000–200,000 yuan	655 (29.4%)	134 (28.6%)	75 (32.3%)	
200,000–300,000 yuan	485 (21.8%)	99 (21.2%)	43 (18.5%)	
300,000–500,000 yuan	443 (19.9%)	107 (22.9%)	42 (18.1%)	
≥500,000 yuan	189 (8.5%)	38 (8.1%)	17 (7.3%)	
Refuse to answer	194 (8.7%)	44 (9.4%)	16 (6.9%)	
Mother’s highest education levels, *n* (%)				
Middle school or below	212 (9.5%)	43 (9.2%)	26 (11.2%)	0.072
High or vocational school	433 (19.4%)	81 (17.3%)	59 (25.4%)	
College or above	1583 (71.1%)	344 (73.5%)	147 (63.4%)	
Father’s highest education levels, *n* (%)				
Middle school or below	159 (7.1%)	30 (6.4%)	19 (8.2%)	0.100
High or vocational school	506 (22.7%)	94 (20.1%)	66 (28.4%)	
College or above	1563 (70.2%)	344 (73.5%)	147 (63.4%)	
Child Physical activity level, *n* (%)				
Inactive	600 (26.9%)	116 (24.8%)	73 (31.5%)	0.003
Insufficiently active	597 (26.8%)	107 (22.9%)	74 (31.9%)	
Sufficiently active	1031 (46.3%)	245 (52.4%)	85 (36.6%)	
Child Dietary pattern				
Total energy intake (kcal), mean (SD)	2884 (1863)	2873 (1806)	2731 (1873)	0.398

Abbreviations: BMI, body mass index; SD, standard deviation. * The *p*-values are the results of comparisons between the children and adolescents classified as having normal weight, overweight, and obesity, using chi-square tests for categorical variables and analysis of variance (ANOVA) for continuous variables.

**Table 2:** Screen use and associations with weight status at baseline.

Screen Use	Total	Overweight			Obesity		
	Mean (SD)	Mean (SD)	*p*-Value ^u^	*p*-Value ^a^	Mean (SD)	*p*-Value ^u^	*p*-Value ^a^
Recreational screen time							
Watching TV/videos	18.1 (25.7)	17.9 (23.6)	0.987	0.826	21.4 (29.7)	0.049	0.038
Computer/smartphone gaming	7.2 (17.3)	7.8 (15.3)	0.121	0.707	10.8 (25.0)	<0.001	0.007
Social media use	10.5 (22.7)	9.5 (19.4)	0.184	0.218	8.8 (22.4)	0.162	0.968
Browsing webpages	5.4 (13.8)	5.7 (12.7)	0.491	0.375	5.7 (16.0)	0.595	0.210
Total	41.3 (58.3)	40.9 (49.4)	0.950	0.950	46.9 (71.7)	0.133	0.040
Educational screen time							
Online homework	7.5 (22.3)	7.4 (22.1)	0.787	0.871	6.8 (13.5)	0.578	0.687
Online class	75.1 (96.4)	75.4 (95.6)	0.890	0.640	68.0 (97.1)	0.228	0.527
Total	82.7 (100.6)	82.9 (98.7)	0.846	0.627	74.7 (100.0)	0.200	0.485

*p*-value ^u^: Unadjusted *p*-value; *p*-value ^a^: *p*-value adjusted for sex and age. The *p*-values are the results of multinomial logistic regression using the normal weight group as the reference group.

**Table 3:** Longitudinal data analysis using mixed effect models studying effects of screen time on obesity.

Parameter	OR (95% CI) _a_	OR (95% CI) _b_	OR(95% CI) _c_
Recreational screen time			
Watching TV/videos	1.565 (1.231–1.989)	1.557 (1.219–1.988)	1.576 (1.230–2.020)
Computer/smartphone gaming	1.081 (0.818–1.427)	1.063 (0.801–1.410)	1.073 (0.811–1.419)
Social media use	1.260 (0.947–1.675)	1.241 (0.930–1.657)	1.265 (0.948–1.686)
Browsing webpages	1.147 (0.905–1.454)	1.148 (0.906–1.455)	1.161 (0.916–1.472)
Total	1.503 (1.155–1.957)	1.487 (1.139–1.943)	1.518 (1.159–1.988)
Educational screen time			
Online homework	1.041 (0.764–1.418)	1.025 (0.757–1.388)	1.032 (0.763–1.395)
Online class	1.114 (0.712–1.744)	1.094 (0.703–1.705)	1.102 (0.707–1.720)
Total	1.115 (0.728–1.707)	1.089 (0.715–1.658)	1.107 (0.726–1.686)

Abbreviations: OR, odds ratio; CI, confidence interval; OR (95% CI) _a_: Adjusting for grade and sex; OR (95% CI) _b_: Adjusting for grade, sex, parental educational levels, and parental BMI; OR (95% CI) _c_: Adjusting for grade, sex, parental educational levels, parental BMI, physical activity level, and total energy intake; Bold lines indicate significant associations (*p* < 0.05). The ORs were computed by contrasting the children and adolescents classified as having obesity to those classified as overweight/normal weight.

**Table 4:** Longitudinal data analysis using mixed effect models studying age-stratified effects of screen time on obesity.

Parameter	OR (95% CI) _a_	OR (95% CI) _b_	OR(95% CI) _c_
Children (*n* = 649)			
Recreational screen time			
Watching TV/videos	1.483 (1.092–2.015)	1.482 (1.079–2.036)	1.489 (0.948–2.337)
Computer/smartphone gaming	1.079 (0.755–1.542)	1.072 (0.742–1.549)	1.008 (0.622–1.634)
Social media use	1.347 (0.949–1.911)	1.316 (0.918–1.886)	1.366 (0.900–2.074)
Browsing webpages	1.129 (0.800–1.594)	1.146 (0.811–1.618)	1.007 (0.716–1.416)
Total	1.452 (1.041–2.026)	1.450 (1.029–2.043)	1.459 (0.907–2.344)
Educational screen time			
Online homework	1.321 (0.952–1.832)	1.283 (0.919–1.791)	0.553 (0.298–1.026)
Online class	1.146 (0.660–1.990)	1.102 (0.640–1.899)	0.912 (0.424–1.963)
Total	1.353 (0.805–2.276)	1.285 (0.769–2.147)	0.646 (0.301–1.387)
Adolescents (*n* = 1579)			
Recreational screen time	1.484 (1.092–2.015)	1.482 (1.079–2.036)	1.485 (1.080–2.042)
Watching TV/videos	1.079 (0.755–1.542)	1.072 (0.742–1.549)	1.071 (0.744–1.542)
Computer/smartphone gaming	1.347 (0.949–1.911)	1.316 (0.918–1.886)	1.332 (0.927–1.914)
Social media use	1.129 (0.800–1.594)	1.146 (0.811–1.618)	1.158 (0.823–1.631)
Browsing webpages	1.452 (1.040–2.026)	1.450 (1.029–2.043)	1.470 (1.040–2.078)
Total			
Educational screen time	1.321 (0.952–1.832)	1.283 (0.919–1.791)	1.274 (0.913–1.778)
Online homework	1.146 (0.660–1.991)	1.102 (0.640–1.899)	1.108 (0.643–1.908)
Online class	1.353 (0.805–2.276)	1.285 (0.769–2.147)	1.287 (0.770–2.151)
Total	1.484 (1.092–2.015)	1.482 (1.079–2.036)	1.485 (1.080–2.042)

Abbreviations: OR, odds ratio; CI, confidence interval; OR (95% CI) _a_: Adjusting for grade and sex; OR (95% CI) _b_: Adjusting for grade, sex, parental educational levels, and parental BMI; OR (95% CI) _c_: Adjusting for grade, sex, parental educational levels, parental BMI, physical activity level, and total energy intake; Bold lines indicate significant associations (*p* < 0.05). The ORs were computed by contrasting the children/adolescents classified as having obesity to those classified as overweight/normal weight.

## Data Availability

The datasets generated during and/or analyzed during the current study are not publicly available due to privacy or ethical restriction but are available from the corresponding author upon reasonable request.

## Supplementary Materials

The following supporting information can be downloaded at: https://www.mdpi.com/article/10.3390/nu15092055/s1, Table S1 Baseline and follow-up measurement dates; Table S2 Longitudinal data analysis using mixed effect models studying effects of screen time on BMI; Table S3 Longitudinal data analysis using mixed effect models studying age-stratified effects of screen time on BMI.
