# Supplementary material for: Association between Types of Screen Time and Weight Status during the COVID-19 Pandemic: A Longitudinal Study in Children and Adolescents
Source: Nutrients. 2023 Apr 24;15(9):2055. doi: 10.3390/nu15092055 (PMC10181058; doi:10.3390/nu15092055)
Supplement: Supplementary file 1 [file nutrients-15-02055-s001.zip › nutrients-2309862-supplementary.pdf]

Table S1 Baseline and follow-up measurement dates

**Date**	**Significant events**
January 3rd to 21st	Baseline survey
January 24th	Level 1 public health emergency response declared
January 25th	The lockdown began
March 2nd	Primary and secondary schools started online classes
March 13nd to 23rd	Follow-up survey
March 24th	Public health emergency response downgraded to level 2
March 25th -	The lockdown relaxed

Table S2 Longitudinal data analysis using mixed effect models studying effects of screen time on BMI

Parameter	Estimate (95%CI)a	Estimate (95%CI)b	Estimate (95%CI)c
**Recreational screen time**			
Watching TV/videos	**0.097 (0.039-0.154)**	**0.091 (0.034-0.148)**	**0.091 (0.033-0.148)**
Computer/smartphone gaming	0.008 (-0.051-0.066)	0.004 (-0.055-0.063)	0.003 (-0.055-0.062)
Social media use	0.027 (-0.032-0.086)	0.025 (-0.034-0.084)	0.025 (-0.034-0.084)
Browsing webpages	0.032 (-0.022-0.087)	0.033 (-0.021-0.087)	0.033 (-0.021-0.087)
Total	**0.077 (0.016-0.138)**	**0.072 (0.011-0.133)**	**0.071 (0.010-0.132)**
**Educational screen time**			
Online homework	-0.022 (-0.077-0.034)	-0.024 (-0.080-0.031)	-0.025 (-0.081-0.030)
Online class	0.009 (-0.058-0.077)	0.005 (-0.062-0.072)	0.006 (-0.061-0.073)
Total	-0.003 (-0.072-0.066)	-0.008 (-0.077-0.061)	-0.008 (-0.076-0.061)

Abbreviations: CI, confidence interval

Estimate (95%CI)a: Adjusting for grade and sex

Estimate (95%CI)b: Adjusting for grade, sex, parental educational levels, and parental BMI

Estimate (95%CI)c: Adjusting for grade, sex, parental educational levels, parental BMI, physical activity level, and total energy intake

Bold lines indicate significant associations ($p<0.05$)

Estimates and CIs were derived from the regression coefficients of linear mixed-effect model

Table S3 Longitudinal data analysis using mixed effect models studying age-stratified effects of screen time on BMI

**Parameter**	**Estimate (95%CI)a**	**Estimate (95%CI)b**	**Estimate (95%CI)c**
	**Children (n = 649)**		
**Recreational screen time**			
Watching TV/videos	0.118 (-0.005-0.242)	0.105 (-0.018-0.228)	0.103 (-0.020-0.227)
Computer/smartphone gaming	0.012 (-0.113-0.138)	-0.002 (-0.128-0.123)	-0.007 (-0.132-0.119)
Social media use	0.035 (-0.083-0.153)	0.034 (-0.084-0.151)	0.035 (-0.083-0.153)
Browsing webpages	0.018 (-0.096-0.132)	0.019 (-0.095-0.134)	0.020 (-0.094-0.135)
Total	0.099 (-0.030-0.224)	0.085 (-0.042-0.211)	0.083 (-0.044-0.210)
**Educational screen time**			
Online homework	-0.052 (-0.172-0.068)	-0.053 (-0.173-0.066)	-0.058 (-0.178-0.063)
Online literature class	0.047 (-0.114-0.210)	0.046 (-0.115-0.208)	0.050 (-0.111-0.212)
Total	0.007 (-0.154-0.168)	0.005 (-0.155-0.166)	0.006 (-0.155-0.167)
	**Adolescents (n = 1579)**		
**Recreational screen time**			
Watching TV/videos	**0.070 (0.007-0.134)**	**0.067 (0.003-0.130)**	**0.066 (0.003-0.130)**
Computer/smartphone gaming	0.011 (-0.053-0.075)	0.009 (-0.055-0.074)	0.008 (-0.056-0.073)
Social media use	0.060 (-0.004-0.124)	0.058 (-0.006-0.122)	0.057 (-0.007-0.121)
Browsing webpages	0.040 (-0.021-0.100)	0.042 (-0.019-0.102)	0.041 (-0.010-0.102)
Total	**0.081 (0.013-0.149)**	**0.078 (0.010-0.145)**	**0.077 (0.009-0.145)**
**Educational screen time**			
Online homework	-0.004 (-0.065-0.057)	-0.006 (-0.067-0.055)	-0.006 (-0.068-0.055)
Online class	-0.004 (-0.074-0.067)	-0.007 (-0.078-0.064)	-0.006 (-0.077-0.065)
Total	-0.005 (-0.078-0.068)	-0.009 (-0.082-0.064)	-0.009 (-0.082-0.064)

Abbreviations: CI, confidence interval
Estimate (95%CI)[a]: Adjusting for grade and sex
Estimate (95%CI)[b]: Adjusting for grade, sex, parental educational levels, and parental BMI
Estimate (95%CI)[c]: Adjusting for grade, sex, parental educational levels, parental BMI, physical activity level, and total energy intake
Bold lines indicate significant associations ($p<0.05$)
Estimates and CIs were derived from the regression coefficients of linear mixed-effect model
